# Construction a new nomogram prognostic model for predicting overall survival after radical resection of esophageal squamous cancer

**DOI:** 10.3389/fonc.2023.1007859

**Published:** 2023-03-21

**Authors:** Bowen Shi, Chunguang Li, Wenqiang Xia, Yuerong Chen, Hezhong Chen, Li Xu, Ming Qin

**Affiliations:** ^1^ Department of Thoracic Surgery, Changhai Hospital, Naval Medical University, Shanghai, China; ^2^ Department of Thoracic Surgery, Shanghai Chest Hospital, Shanghai Jiaotong University, Shanghai, China; ^3^ Department of General Surgery, Tengchong People’s Hospital, Tengchong, China; ^4^ Department of Thoracic Surgery, Shanghai Pulmonary Hospital, Shanghai Tongji University, Shanghai, China; ^5^ School of Basic Medicine, Naval Medical University, Shanghai, China

**Keywords:** esophageal squamous cell carcinoma, prognostic model, nomogram, TNM staging system, Cox regression

## Abstract

**Background:**

Esophageal cancer is one of the deadliest malignancies in the world, and 5-year overall survival (OS) of esophageal cancer ranges from 12% to 20%. Surgical resection remains the principal treatment. The American Joint Commission on Cancer (AJCC) TNM (tumor, node, and metastasis) staging system is a key guideline for prognosis and treatment decisions, but it cannot fully predict outcomes. Therefore, targeting the molecular and biological features of each patient’s tumor, and identifying key prognostic biomarkers as effective survival predictors and therapeutic targets are highly important to clinicians and patients.

**Methods:**

In this study, three different methods, including Univariate Cox regression, Lasso regression, and Randomforest regression were used to screen the independent factors affecting the prognosis of esophageal squamous cell carcinoma and construct a nomogram prognostic model. The accuracy of the model was verified by comparing with TNM staging system and the reliability of the model was verified by internal cross validation.

**Results:**

Preoperative neutrophil lymphocyte ratio(preNLR), N-stage, p53 level and tumor diameter were selected to construct the new prognostic model. Patients with higher preNLR level, higher N-stage, lower p53 level and larger tumor diameter had worse OS. The results of C-index, Decision Curve Analysis (DCA), and integrated discrimination improvement (IDI) showed that the new prognostic model has a better prediction than the TNM staging system.

**Conclusion:**

The accuracy and reliability of the nomogram prognostic model were higher than that of TNM staging system. It can effectively predict individual OS and provide theoretical basis for clinical decision making.

## 1 Introduction

Esophageal cancer is one of the deadliest malignancies in the world and has a poor prognosis (1). Its major subtypes are esophageal squamous cell carcinoma and adenocarcinoma (2). Esophageal squamous cell carcinoma accounts for 70% of cases of esophageal cancer globally. Esophageal cancer is the fourth most common cancer in China (3) and approximately 70% of global esophageal cancer cases occur in China (4). Although the incidence of esophageal cancer in China has declined in recent years, the absolute number of patients is still high due to the large population.

At present, radical operation is the most effective strategy for the treatment of early esophageal squamous cell carcinoma and the best choice for long-term survival of esophageal squamous cell carcinoma patients. In patients with advanced tumors, a combination of preoperative and perioperative chemoradiotherapy is often required, but the results are still unsatisfactory. The 5-year survival rate for patients undergoing radical resection of esophageal cancer is only 13% to 18% (5).

At present, the AJCC TNM staging system is mainly used to evaluate the postoperative prognosis of esophageal cancer. However, due to individual differences, some patients with the same TNM stage may have different prognosis even after receiving the same treatment (6).

In addition to TNM staging system, studies have shown that other patient characteristics may also affect the prognosis after radical resection of esophageal cancer (7–10). Therefore, in this study, a new prognostic model for patients undergoing esophageal cancer radical operation was established based on the characteristics of patients by collecting and analyzing clinical data. The results showed that the accuracy and reliability of the new nomogram prognostic model is better than that of TNM model. It is of great value to predict the overall survival of esophageal squamous cell carcinoma patients after radical operation.

## 2 Materials and methods

### 2.1 General patient information

The clinical data of 256 patients with esophageal squamous cell carcinoma undergoing radical resection were retrospectively analyzed in the Department of Thoracic surgery of Changhai Hospital Affiliated to Naval Medical University from November 2015 to October 2017.

Inclusion criteria (1): radical surgical indications (2); postoperative pathological diagnosis was esophageal squamous cell carcinoma (3); complete clinical data and follow-up data (4). did not receive any antitumor therapy before surgical esophagectomy.

Exclusion criteria (1): complicated with other malignancies (2); overall survival <3 months (3); with severe complications after surgery.

This study was approved by the Hospital Ethics Review Committee.

### 2.2 Pathological examination results

The pathological staging of esophageal squamous cell carcinoma was conducted according to the 8^th^ edition American Joint Commission on Cancer (AJCC) staging system. Patients’ T-stage, N-stage and P-stage were determined by experienced clinicians and pathologists based on pathological examination results. Immunohistochemistry of LEF1, Ki67 and p53 were detected. Tissues were embedded in paraffin and analyzed by the avidin-biotin complex method. Immunohistochemical results were scored by two experienced pathologists who were unaware of clinical and follow-up information.

### 2.3 Study endpoints and information collection

The endpoint of the study was overall survival (OS) of patients. OS was defined as the time from surgery to death or the last follow-up. All patients were followed up periodically by telephone, with the last follow-up date being July 2022.

Peripheral blood biochemical information was collected within one week before surgery. Preoperative neutrophil lymphocyte ratio(preNLR) is equal to the absolute number of neutrophils divided by the absolute number of lymphocytes.

### 2.4 Statistical analysis

As there was no unified and verified cut-off value, the cut-off value of continuous variables such as preNLR, p53, Ki-67 and hospital stay were calculated by using the Log-rank test based on Kaplan-Meier curve, which made the data on both sides of the cut-off value have the best difference.

Three variable screening methods were used to screen variables in the new prognostic model (1): Univariate Cox regression: Based on the results of univariate Cox regression, variables with significant differences (P <0.05) were included in the multivariate Cox regression model (2). Lasso regression: Lasso regression used the “glmnet” package to screen the best combination of variables. In order to select the model with excellent performance and the least number of independent variables, we set lambda (λ)=lambda.1se (3). Randomforest regression: RandomForest regression filtered variables through “randomForest” package, and set parameters as ntree=200, mtry=8, sampsize=100. Then the max-subtree function was used to screen out variables with high conservatism.

The selected variables were incorporated into the multivariate Cox regression model and a nomogram was constructed using the “rms” package to visualize the results of multivariate Cox regression model. This nomogram can convert the correlation coefficients of the Cox proportional risk model into 0-100 points to calculate the total score. Then the 1-year, 3-year, and 5-year OS rates were obtained according to the total score. ROC curve was used to compare the prognostic models, and AUC was used to evaluate the best model. The nomogram prognostic model was verified internally using “bootstrap” package to calculate the C-index of the model. Finally, the DCA and IDI was used to evaluate the benefits of nomogram model.

The above analyses were implemented using R language (version 4.1.2).

The relationship between preNLR and other clinical characteristics in this study was analyzed by Pearson χ2 test. P < 0.05 was considered statistically significant. The above analyses were implemented using SPSS Statistic (version 25).

## 3 Results

### 3.1 Patients and tumor baseline characteristics

**Figure 1:**
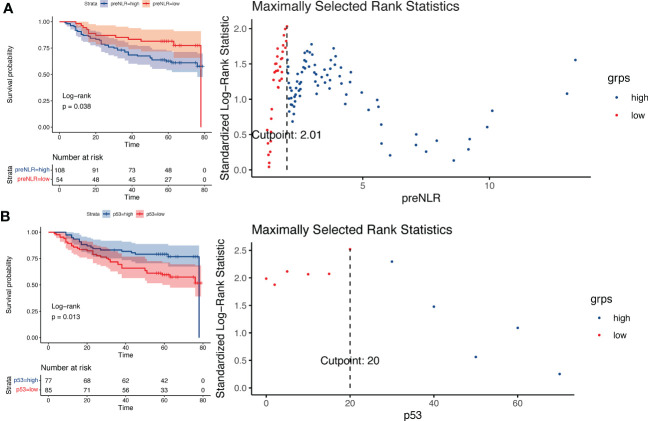
Log-rank test based on Kaplan-Meier curve. **(A)** Kaplan-Meier curve of different preNLR level groups and risk coefficients in different periods, cut-off value=2.01. **(B)** Kaplan-Meier curve of different p53 level groups and risk coefficients in different periods, cut-off value=20%.

This study included 256 patients with esophageal squamous cell carcinoma undergoing radical resection. The baseline characteristics were shown in **Table 1**. We divided patients into a training cohort and a validation cohort by random grouping, including 160 in the training cohort and 96 in the validation cohort. Then we constructed a prognostic model in the training cohort and verified the reliability and accuracy of the model in the validation cohort. According to the Kaplan-Meier curve, the cut-off value of preNLR is 2.01(**Figure 1A**), the cut-off value of p53 is 20% (**Figure 1B**), the cut-off value of Ki-67 is 69% and the cut-off value of hospital stay is 15 days (**Supplementary Figure 1**) in the training cohort.

**Table 1:** General patient information.

Variables	No. (%)
preNLR	
<=2.01	89 (34.8%)
>2.01	167 (65.2%)
Gender	
Male	72 (28.2%)
Female	184 (71.8%)
Age	
<=65	156 (60.9%)
>65	100 (39.1%)
BMI	
<=24	160 (62.5%)
>24	96 (37.5%)
Tumer diameter	
<=3cm	140 (54.7%)
>3cm	116 (45.3%)
Tumor location	
Upper	39 (15.2%)
Middle	161 (62.9%)
Lower	56 (21.9%)
T-stage	
T1	82 (32.0%)
T2	75 (29.3%)
T3	94 (36.7%)
T4	5 (2.0%)
N-stage	
N0	152 (59.4%)
N1	64 (25.0%)
N2	27 (10.5%)
N3	13 (5.1%)
P-stage	
P0	10 (3.9%)
P1	68 (26.6%)
P2	103 (40.2%)
P3	72 (28.1%)
P4	3 (1.2%)
p53	
>20%	111 (43.4%)
<=20%	145 (56.6%)
Ki67	
>69%	91 (35.5%)
<=69%	165 (64.5%)
LEF1	
High	147 (57.5%)
Low	109 (42.5%)
Hypertension	
Yes	79 (30.9%)
None	177 (69.1%)
COPD	
Yes	2 (0.8%)
None	254 (99.2%)
Diabetes	
Yes	14 (5.5%)
None	242 (94.5%)
Smoke	
Yes	122 (47.7%)
None	134 (52.3%)
Alcohol	
Yes	83 (32.4%)
None	173 (67.6%)
Hospital stay	
≤15d	193 (75.4%)
>15d	63 (24.6%)
Complications	
Yes	85 (33.2%)
None	171 (66.8%)
Hydrothorax caused by surgery	
Yes	37 (14.5%)
None	219 (85.5%)
Bleeding	
Yes	24 (9.4%)
None	232 (90.6%)
Anastomotic_fistula	
Yes	12 (4.7%)
None	244 (95.3%)
Hydrothorax	
Yes	45 (17.6%)
None	211 (82.4%)

### 3.2 Screening of variables in the prognostic model

#### 3.2.1 Univariate Cox regression

Univariate Cox regression analysis showed that tumor diameter, tumor location, T-stage, N-stage, preNLR level, p53 level, LEF1 level were correlated with the OS of patients undergoing radical operation (P < 0.05). The results were shown in **Table 2**. Variables with P < 0.05 were included in multivariate Cox regression.

**Table 2:** Univariate Cox regression for OS.

Variables	P-value	HR (95% CI)
preNLR		
<=2.01	1	
>2.01	0.034	2 (1.05-3.8)
Hospital stay		
<=15d	1	
>15d	0.11	0.5 (0.21-1.17)
p53		
<=20%	1	
>20%	0.011	0.48 (0.27-0.84)
Ki67		
<=69%	1	
>69%	0.14	1.5 (0.88-2.56)
Gender		
Female	1	
Male	0.716	1.12 (0.61-2.06)
Age		
<=65	1	
>65	0.594	1.16 (0.67-1.99)
BMI		
<=24	1	
>24	0.327	0.75 (0.43-1.33)
Hypertension		
None	1	
Yes	0.677	0.88 (0.49-1.59)
COPD		
None	1	
Yes	0.995	0 (0-Inf)
Diabetes		
None	1	
Yes	0.258	0.32 (0.04-2.31)
Smoke		
None	1	
Yes	0.366	1.28 (0.75-2.2)
Alcohol		
None	1	
Yes	0.122	1.54 (0.89-2.65)
Tumor location		
Upper	1	
Middle	0.039	0.43 (0.19-0.96)
Lower	0.656	0.83 (0.36-1.89)
Tumer diameter		
<=3cm	1	
>3cm	<0.001	3.64 (2.02-6.53)
T-stage		
T1	1	
T2	0.008	3.42 (1.38-8.47)
T3	<0.001	5.54 (2.43-12.63)
T4	0.028	5.83 (1.21-28.12)
N-stage		
N0	1	
N1	<0.001	3.5 (1.76-6.96)
N2	<0.001	5.89 (2.8-12.4)
N3	<0.001	13.85 (5.58-34.34)
P-stage		
P0	1	
P1	0.996	12205051.48 (0-Inf)
P2	0.996	33547535.29 (0-Inf)
P3	0.995	106852798.93 (0-Inf)
P4	0.995	84680562.26 (0-Inf)
LEF1		
Low	1	
High	0.034	1.88 (1.05-3.39)
Complications		
None	1	
Yes	0.373	0.77 (0.44-1.36)
Hydrothorax caused by surgery		
None	1	
Yes	0.649	1.2 (0.54-2.66)
Bleeding		
None	1	
Yes	0.777	1.13 (0.48-2.65)
Anastomotic.fistula		
None	1	
Yes	0.85	1.11 (0.39-3.16)
Hydrothorax		
None	1	
Yes	0.108	0.56 (0.27-1.14)

#### 3.2.2 Lasso regression

**Figure 2:**
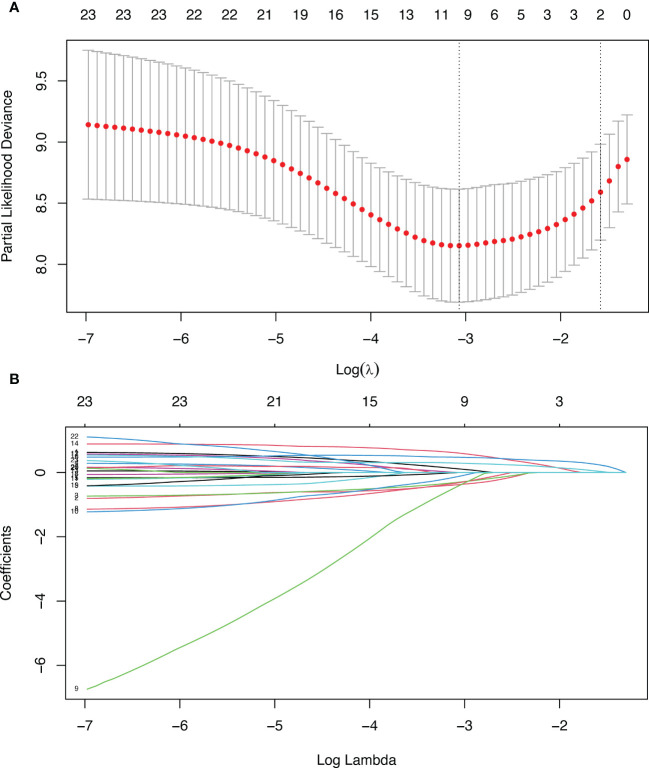
Lasso regression. **(A)** Penalty parameter diagram, Partial-likelihood deviance with Log(λ). Vertical line on the left represents λ=lambda.min, vertical line on the right represents λ=lambda.1se **(B)** The coefficients of different variables vary with the punishment of λ.

In Lasso regression, the variable lambda (λ) was introduced to find the best prognostic model, and λ determined which variables made the model optimal. The advantage of Lasso regression was that it solves the problem of collinearity between variables. When λ=lambda.1se (**Figure 2A**), a model with good performance and minimum number of independent variables was obtained. Therefore, in this study, we chose this value. When λ=lambda.1se, two variables (N-stage and P-stage) were included in the prognostic model (**Figure 2B**). Therefore, N-stage and P-stage were included in multivariate Cox regression.

#### 3.2.3 Randomforest regression

**Figure 3:**
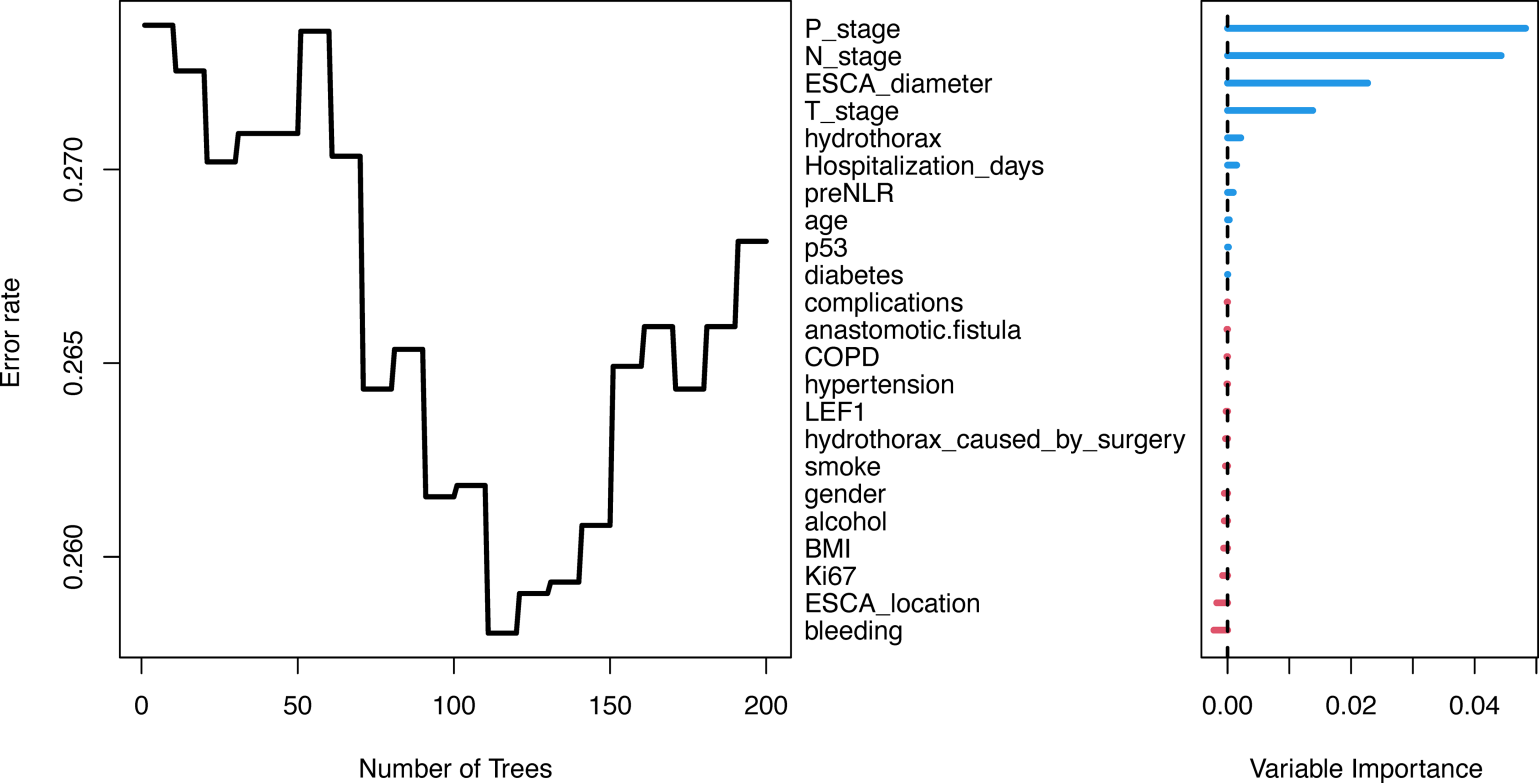
Randomforest regression. Variable importance rank by Randomforest regression.

Randomforest regression is commonly used to evaluate the importance of variables and has good predictive accuracy. In this study, Bootstrap autonomous sampling method was used to randomly select 200 sample sets (samplesize =100) that were put back into the original data set to form 200 decision trees. Combined with the decision results of 200 trees, the importance of variables was comprehensively evaluated. The variables were ranked as shown in **Figure 3**. Then, max-subtree function was used to screen out the first several variables with high conservatism, including tumor diameter, N-stage, P-stage and T-stage. Therefore, tumor diameter, N-stage, P-stage and T-stage were included in multivariate Cox regression.

### 3.3 Construction of nomogram prognostic models

Seven variables with P < 0.05 were screened by univariate Cox regression, two by Lasso regression, and four by Randomforest regression. The variable combinations screened by three methods were incorporated into the multivariate Cox regression model (by backward method). The final models of the three methods were determined by the minimum Akaike information criterion (AIC) value.

In the multivariate Cox regression model, preNLR, N-stage, p53 and tumor diameter were reserved in variables screened by univariate Cox regression, AIC=469.81.

N-stage and P-stage were reserved in variables screened by Lasso regression, AIC=476.18.

Tumor diameter, N-stage and P-stage were reserved in variables screened by Randomforest regression, AIC=470.9172.

Among them, the model screened by univariate Cox regression had the lowest AIC value.

**Figure 4:**
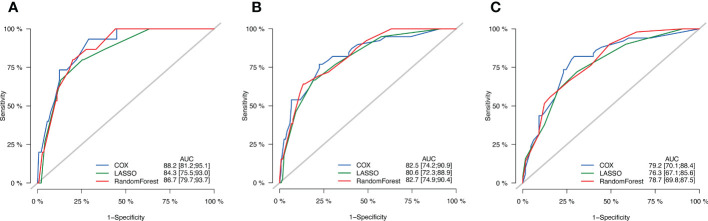
ROC curves of three models. **(A)** ROC curves of 1-year OS. **(B)** ROC curves of 3-year OS. **(C)** ROC curves of 5-year OS.

In order to compare these three models, ROC curve and AUC value were used to evaluate the prognostic models (**Figure 4**). The results showed that the AUC value of the prognostic model screened by univariate Cox regression were the highest when predicting the 1-year and 5-year OS probability, and there was little difference between the prognostic model screened by univariate Cox regression and Randomforest regression when predicting the 3-year OS probability. Therefore, the prognostic model screened by univariate Cox regression was finally adopted, and the variable combinations were preNLR, N-stage, p53 and tumor diameter.

In order to better display the results of multivariate Cox regression, this study introduced the nomogram. Nomogram is widely used for cancer prognosis and has the advantage of quantifying the contribution of variables in prognostic models into estimates of event probabilities. It could provide reference for clinical decision making and screening of high-risk patients.

**Figure 5:**
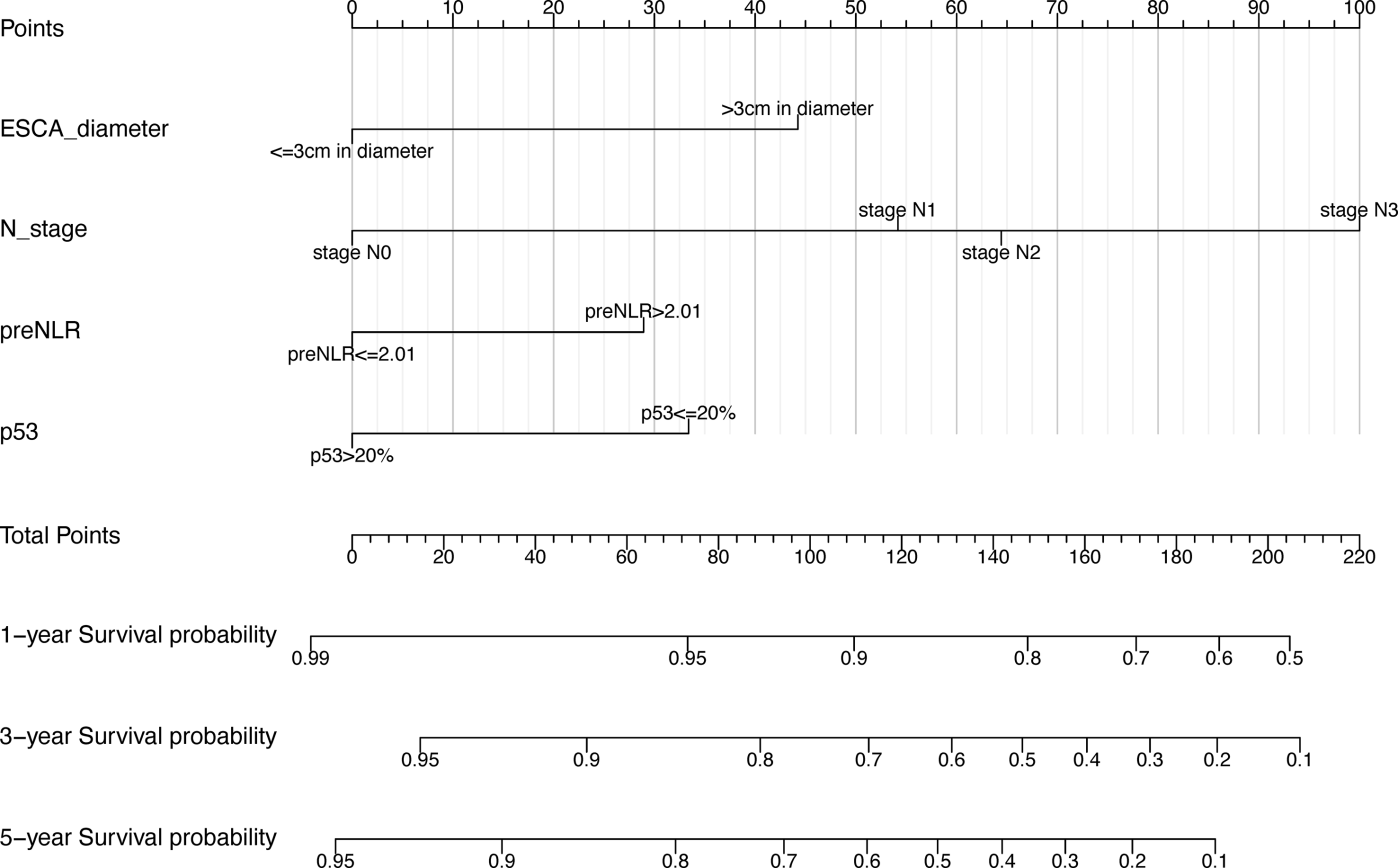
Nomogram. Nomogram prognostic model based on the best multivariate Cox regression model.

Based on the independent prognostic factors screened out above, a nomogram prognostic model of 1-year, 3-year, and 5-year postoperative OS rates for esophageal squamous cell carcinoma patients was constructed using R language (version 4.1.2). In our nomogram prognostic model, tumor diameter >3cm, preNLR>2.01, p53<=20% and higher N-stage were independent prognostic factors for esophageal squamous cell carcinoma patients (**Figure 5**). The model had important value in predicting postoperative overall survival of patients undergoing radical resection of esophageal squamous cell carcinoma.

### 3.4 Validation of nomogram prognostic models

**Figure 6:**
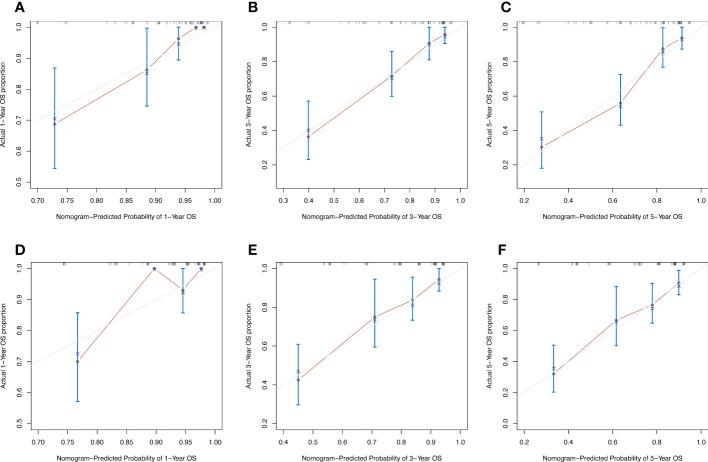
Calibration curves of 1-year, 3-year and 5-year OS predicted by two models. **(A–C)** Calibration curves of new nomogram prognostic model. **(D–F)** Calibration curves of TNM prognostic model.

In order to compare the advantages and disadvantages between the new prognostic model and the traditional TNM prognostic model, the C-index was generated through internal cross validation. The results showed that the C-index of the new prognostic model was 0.785 (95%CI 0.662-0.908), and that of the TNM prognostic model was 0.765 (95%CI 0.636-0.894). The predictive accuracy of the new prognostic model is better than that of TNM model. The calibration curves of 1-year, 3-year and 5-year overall survival predicted by the new prognostic model (**Figures 6A–C**) and the TNM prognostic model (**Figure 6D–F**) were shown in **Figure 6**. The results also showed that the prediction accuracy of the new prognostic model was slightly better than that of the TNM prognostic model.

**Figure 7:**
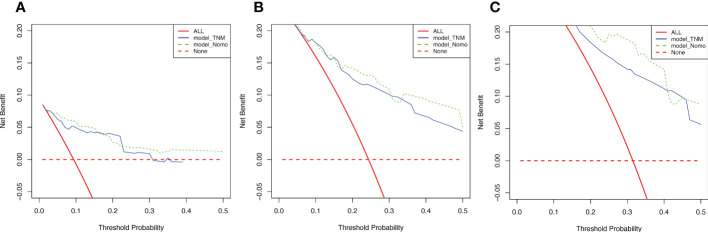
Decision curves of two models. **(A)** Decision curves of 1-year OS. **(B)** Decision curves of 3-year OS. **(C)** Decision curves of 5-year OS.

**Figure 8:**
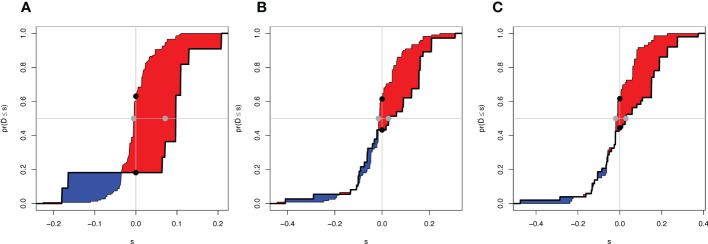
Integrated Discrimination Improvement of two models. **(A)** IDI of 1-year OS. **(B)** IDI of 3-year OS. **(C)** IDI of 5-year OS. The difference between the red area and the blue area is IDI.

To evaluate the reliability of nomogram prognostic model for postoperative 1-year, 3-year, and 5-year overall survival, we use Decision Curve Analysis (DCA) at different decision thresholds (**Figures 7A–C**). The results showed that the prediction line of the new prognosis model was higher than that of the TNM prognosis model. Further, we use integrated discrimination improvement (IDI) to evaluate the reliability of nomogram prognostic model. The results showed that compared with the TNM prognosis model, the prediction probability of our new nomogram prognostic model improved by 6.0% (P =0.040) at the first year, 4.2% (P =0.079) at the third year, and 5.4% (P =0.048) at the fifth year (**Figures 8A–C**).

At last, we verified our new nomogram prognostic model in the validation cohort. The results showed that the C-index of the new prognostic model in the validation cohort was 0.773 (95%CI 0.675-0.871). Further, we use integrated discrimination improvement (IDI) to evaluate the reliability of nomogram prognostic model. The results showed that compared with the TNM prognosis model, the prediction probability of our new nomogram prognostic model improved by 3.7% at the fifth year. Therefore, the new prognostic model showed a better prediction of postoperative death for esophageal squamous cell carcinoma.

### 3.5 Relationship between preNLR and other baseline characteristics

At last, this study compared the clinical characteristics of esophageal cancer patients at different NLR levels (**Table 3**). The results showed that compared with preNLR<=2.01 group, preNLR>2.01 group had a higher T stage (χ2 = 11.315, P =0.004). Cramer’s V value between preNLR and T stage was 0.210, P=0.004, indicating that there was a linear correlation between preNLR and T stage. With the increase of T stage, preNLR>2.01 proportion became higher and higher. The results also showed that compared with preNLR<=2.01 group, preNLR>2.01 group had a larger tumor diameter (χ2 = 18.531, P <0.001). Cramer’s V value between preNLR and tumor diameter was 0.269, P <0.001, indicating that there was a linear correlation between preNLR and tumor diameter. With the increase of tumor diameter, preNLR>2.01 proportion became higher and higher.

**Table 3:** Clinical characteristics of patients with different preNLR levels.

Variables	preNLR≤2.01 (n=89)	preNLR>2.01 (n=167)	χ2-value	P-value
Gender				
Male	64 (34.8%)	120 (65.2%)	0.470	0.493
Female	25 (30.5%)	57 (69.5%)		
Age				
<=65	57 (36.5%)	99 (63.5%)	0.553	0.457
>65	32 (32.0%)	68 (68.0%)		
BMI				
<=24	58 (36.3%)	102 (63.7%)	0.415	0.520
>24	31 (32.3%)	65 (67.7%)		
Tumor diameter				
<=3cm	65 (46.4%)	75 (53.6%)	18.531	<0.001
>3cm	24 (20.7%)	92 (79.3%)		
Tumor location				
Upper	15 (38.5%)	24 (61.5%)	2.365	0.306
Middle	57 (35.4%)	104 (64.6%)		
Lower	13 (25.0%)	39 (75.0%)		
T-stage				
T1	40 (48.8%)	42 (51.2%)	11.315	0.004
T2	24 (32.0%)	51 (68.0%)		
T3+T4	25 (25.3%)	74 (74.7%)		
N-stage				
N0	57 (37.5%)	95 (62.5%)	3.193	0.203
N1	23 (35.9%)	41 (64.1%)		
N2+N3	9 (22.5%)	31 (77.5%)		
P-stage				
P0	5 (50%)	5 (50%)	6.616	0.085
P1	30 (44.1%)	38 (55.9%)		
P2	35 (34.0%)	68 (66.0%)		
P3+P4	19 (25.3%)	56 (74.7%)		
p53				
>20%	35 (31.5%)	76 (68.5%)	0.904	0.342
<=20%	54 (37.2%)	91 (62.8%)		
Ki67				
>69%	26 (28.6%)	65 (71.4%)	2.389	0.122
<=69%	63 (38.2%)	102 (61.8%)		
Hypertension				
Yes	26 (32.9%)	53 (67.1%)	0.173	0.677
None	63 (35.6%)	114 (64.4%)		
COPD				
Yes	1 (50%)	1 (50%)	0	1
None	88 (34.8%)	166 (65.2%)		
Diabetes				
Yes	4 (28.6%)	10 (71.4%)	0.251	0.617
None	85 (35.1%)	157 (64.9%)		
Smoke				
Yes	37 (30.3%)	85 (69.7%)	2.024	0.155
None	52 (38.8%)	82 (61.2%)		
Alcohol				
Yes	27 (32.5%)	56 (67.5%)	0.271	0.603
None	62 (35.8%)	111 (64.2%)		
Hospital stay				
<=15d	62 (32.1%)	131 (67.9%)	2.412	0.120
>15d	27 (42.9%)	36 (57.1%)		
Complications				
Yes	28 (32.9%)	57 (67.1%)	0.187	0.666
None	61 (35.7%)	110 (64.3%)		
Hydrothorax caused by surgery				
Yes	13 (35.1%)	24 (64.9%)	0.003	0.959
None	76 (34.7%)	143 (65.3%)		
Bleeding				
Yes	7 (29.2%)	17 (70.8%)	0.366	0.545
None	82 (35.3%)	150 (64.7%)		
Anastomotic_fistula				
Yes	4 (33.3%)	8 (66.7%)	0	1
None	85 (34.8%)	159 (65.2%)		
Hydrothorax				
Yes	18 (40.0%)	27 (60.0%)	1.041	0.308
None	71 (32.1%)	150 (67.9%)		

There were no significant differences in other characteristics (P>0.05).

## 4 Discussion

Although up to now, significant progress has been made in the treatment of esophageal squamous cell carcinoma including radical surgery, chemotherapy and radiotherapy, the 5-year overall survival rate is still very low (11–13). The TNM staging system commonly used in clinical practice can only partially predict the prognosis of patients. It has been reported that patients with the same TNM stage may have different prognosis after receiving the same treatment. Therefore, it is of great significance to construct an individualized and accurate prognostic model for clinical judgment of prognosis and adjuvant treatment.

In this study, the clinical characteristics of patients were comprehensively evaluated by univariate Cox regression, Lasso regression and Randomforest regression. Three different multivariate Cox regression models were constructed to effectively avoid the deviation caused by single screening method. The results showed that the AUC value of the prognostic model constructed by univariate Cox regression was the largest. Therefore, it was selected as our prognostic model. In the newly constructed prognostic model, preNLR, N-stage, p53 and tumor diameter were independent factors affecting the prognosis of esophageal squamous cell carcinoma patients after radical operation.

NLR is one of the main indicators of systemic inflammation. According to reports, elevated NLR is a valuable predictor of many cancers, including pancreatic cancer, gastric cancer, breast cancer and so on (14–16). The relationship between NLR and prognosis has also been reported in esophageal cancer (17, 18). However, there is no unified and verified cut-off value for NLR in esophageal cancer. In this study, the cut-off value of NLR is determined to be 2.01 by using the Log-rank test based on Kaplan-Meier curve. Higher preNLR levels were associated with poorer prognosis (P =0.038). Patients were divided into preNLR>2.01 group and preNLR<=2.01 group, and then the clinical characteristics of the two groups were analyzed. The results showed that preNLR level was positively correlated with tumer diameter (P<0.001) and T-stage (P =0.034), and preNLR>2.01 group had larger tumor diameter and higher T stage. There were no significant differences in other clinical characteristics between the two groups.

Much evidence has proved that N-stage significantly affects the prognosis of esophageal cancer (19, 20). Survival rate of patients with low expression of p53 is significantly lower than that of patients with high expression of p53, and the recurrence rate of tumor is significantly higher (21, 22). It has also been reported that the maximum diameter of tumor is an independent factor affecting OS of esophageal cancer (23, 24). The above results are consistent with the results of our study. In this study, N-stage, p53 level and tumor diameter were all independent factors affecting tumor prognosis.

At present, there are few prognostic models for long-term survival of esophageal squamous cell carcinoma patients, and most studies only include a single factor, such as serum inflammatory markers (25), immune genes (26) and nutritional risk index (27). The new nomogram prognostic model designed in this study covered the TNM staging system, inflammatory markers and immunohistochemical information of the tumor, and showed better reliability and accuracy compared with those single-factor prognostic models.

To verify the accuracy and reliability of the new model, we generated the C-index through internal cross validation. The results showed that the C-index of the new model (0.785) was higher than that of TNM staging system (0.765), indicating that the predictive accuracy of the new model was higher than that of TNM staging system. At the same time, the DCA and IDI results also showed that the reliability of the prediction results of the new model was higher than that of the TNM staging system, which showed a good prediction of the disease. In the validation cohort, the results also showed that our new prognostic model had sufficient reliability and accuracy. In conclusion, the new prognostic model constructed in this study has good predictive ability and important guiding significance for the prognosis and treatment of esophageal squamous cell carcinoma patients.

Neoadjuvant chemoradiotherapy is currently used in patients with locally advanced esophageal cancer. This study only involved patients with esophageal cancer who could undergo surgery in the early and middle stages. Therefore, the factor of neoadjuvant chemoradiotherapy has not been included. At the same time, since this study was a retrospective study and the number of patients included was too small, the selection bias of the object could not be avoided. Meanwhile, since this study was a single-center retrospective study, we only performed internal validation of the prognostic model. In the future, we will conduct further external validation of the model or verify our model through prospective clinical trials. And with the advancement of technology and treatment, we will gradually improve our prognostic model.

## 5 Conclusion

After the screening of three algorithms, preNLR level, N-stage, p53 level and tumor diameter were identified as independent factors affecting the prognosis of esophageal squamous cell carcinoma patients. In this study, the new nomogram prognostic model was more accurate and reliable than the TNM staging system. It could effectively predict the overall survival and provide theoretical basis for clinical decision-making and treatment.

## Data availability statement

The raw data supporting the conclusions of this article will be made available by the authors, without undue reservation.

## Ethics statement

The studies involving human participants were reviewed and approved by Shanghai Changhai Hospital Ethics Review Committee. The patients/participants provided their written informed consent to participate in this study. Written informed consent was obtained from the individual(s) for the publication of any potentially identifiable images or data included in this article.

## Author contributions

BS, HC, and CL performed the operation and provided guidance for the idea of the article. MQ and WX analyzed the data and constructed the model. LX and YC collected the data, analyzed the data, and wrote the article. All authors contributed to the article and approved the submitted version.

## Supplementary material

Supplementary Figure 1 Log-rank test based on Kaplan-Meier curve. **(A)** Kaplan-Meier curve of different hospital stay groups and risk coefficients in different periods, cut-off value=15 days. **(B)** Kaplan-Meier curve of different Ki67 level groups and risk coefficients in different periods, cut-off value=69%.
