# Supplementary figures and images for: Construction a new nomogram prognostic model for predicting overall survival after radical resection of esophageal squamous cancer
Source: Front Oncol. 2023 Mar 21;13:1007859. doi: 10.3389/fonc.2023.1007859 (PMC10070853; doi:10.3389/fonc.2023.1007859)

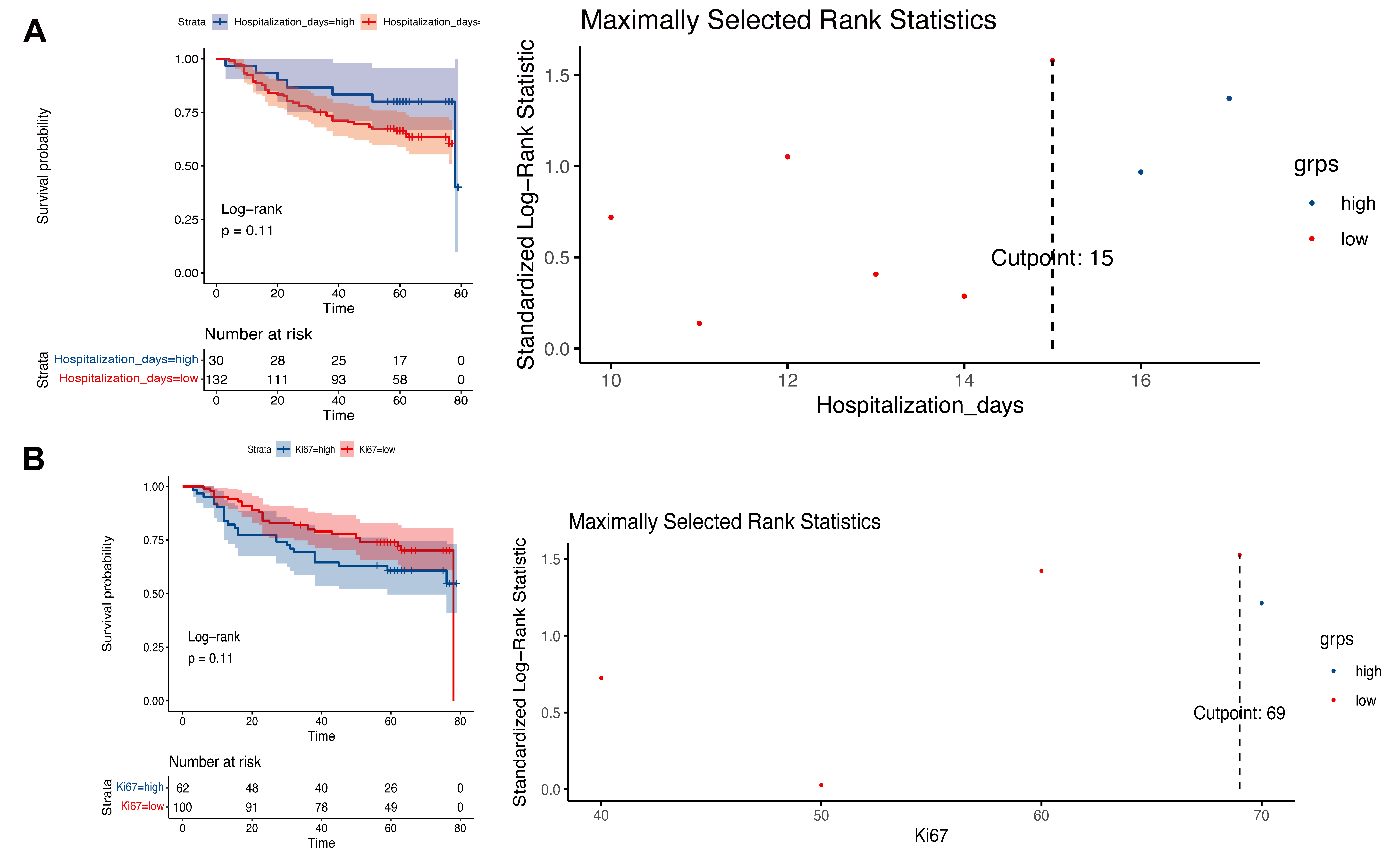

Supplement: Supplementary Figure 1 — Log-rank test based on Kaplan-Meier curve. (A) Kaplan-Meier curve of different hospital stay groups and risk coefficients in different periods, cut-off value=15 days. (B) Kaplan-Meier curve of different Ki67 level groups and risk coefficients in different periods, cut-off value=69%. [file Image_1.tiff]
